# Data on the surface morphology of additively manufactured Ti-6Al-4V implants during processing by plasma electrolytic oxidation

**DOI:** 10.1016/j.dib.2017.06.015

**Published:** 2017-06-09

**Authors:** Ingmar A.J. van Hengel, Martijn Riool, Lidy E. Fratila-Apachitei, Janneke Witte-Bouma, Eric Farrell, Amir A. Zadpoor, Sebastian A.J. Zaat, Iulian Apachitei

**Affiliations:** a Department of Biomechanical Engineering, Faculty of Mechanical, Maritime, and Materials Engineering, Delft University of Technology (TU Delft), Mekelweg 2, 2628 CD Delft, The Netherlands; b Additive Manufacturing Lab, Faculty of Mechanical, Maritime, and Materials Engineering, Delft University of Technology (TU Delft), Mekelweg 2, 2628 CD Delft, The Netherlands; c Department of Medical Microbiology, Center for Infection and Immunity Amsterdam (CINIMA), Academic Medical Center, University of Amsterdam, Meibergdreef 9, 1105 AZ Amsterdam, The Netherlands; d Department of Oral and Maxillofacial Surgery, Special Dental Care and Orthodontics, Erasmus MC, University Medical Centre, Wytemaweg 80, 3015 CN Rotterdam, The Netherlands

**Keywords:** Additive manufacturing, Plasma electrolytic oxidation, Surface morphology, Ti-6Al-4V implants

## Abstract

Additively manufactured Ti-6Al-4V implants were biofunctionalized using plasma electrolytic oxidation. At various time points during this process scanning electron microscopy imaging was performed to analyze the surface morphology (van Hengel et al., 2017) [1]. This data shows the changes in surface morphology during plasma electrolytic oxidation. Data presented in this article are related to the research article “Selective laser melting porous metallic implants with immobilized silver nanoparticles kill and prevent biofilm formation by methicillin-resistant Staphylococcus aureus” (van Hengel et al., 2017) [1].

**Specifications Table**

**Table:** 

Subject area	*Material Science Engineering*
More specific subject area	*Surface biofunctionalization*
Type of data	*Microscopy images*
How data was acquired	*Scanning electron microscope*
Data format	*Raw*
Experimental factors	*Ti-6Al-4V implants were fabricated with selective laser melting. Plasma electrolytic oxidation was performed on a custom-made laboratory setup.*
Experimental features	*Scanning electron microscopy was performed on the surfaces of Ti-6Al-4V implants at various time points during plasma electrolytic oxidation to show the changes in surface morphology.*
Data source location	*Department of Biomechanical Engineering, Faculty of Mechanical, Maritime, and Materials Engineering, Delft University of Technology (TU Delft)*
Data accessibility	*Data is within this article*

**Value of the data**

• Data show the changes in surface morphology of additively manufactured Ti-6Al-4V biomaterials during the plasma electrolytic oxidation process.
• The presented data may be used to compare additively manufactured biomaterials with conventionally produced biomaterials after biofunctionalization by plasma electrolytic oxidation.

• Data may be used to compare the effect of plasma electrolytic oxidation treatment with other biofunctionalization techniques on the surface morphology of additively manufactured Ti-6Al-4V biomaterials.

## 1 Data

**Fig. 1:**
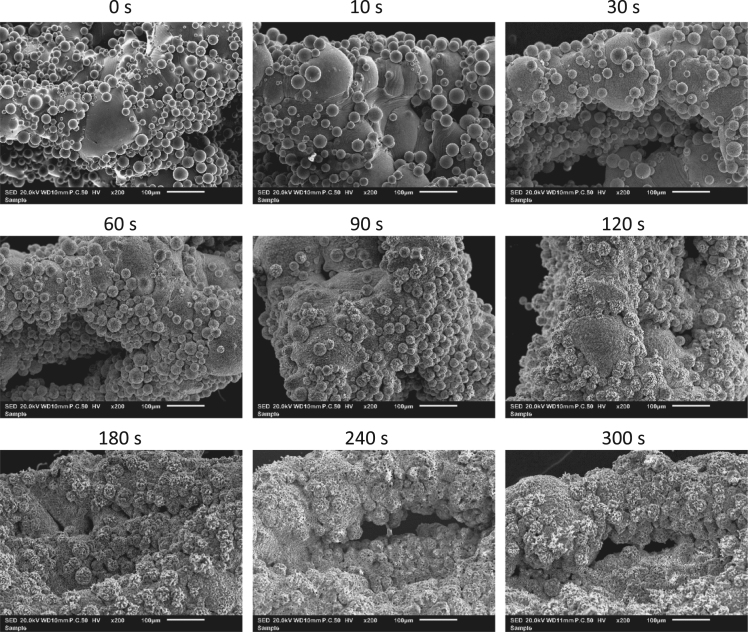
200-fold magnification scanning electron microscopy images at different time points of Ti-6Al-4V implants treated with plasma electrolytic oxidation.

**Fig. 2:**
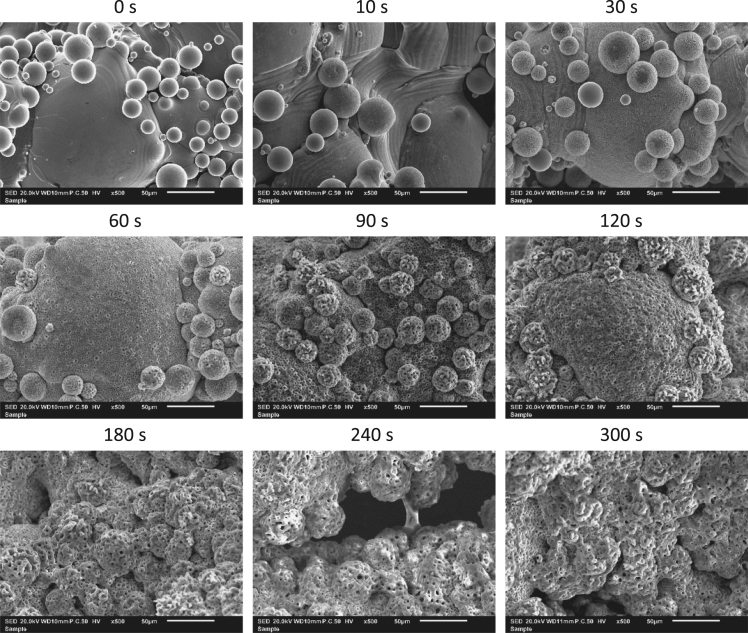
500-fold magnification scanning electron microscopy images at different time points of Ti-6Al-4V implants treated with plasma electrolytic oxidation.

**Fig. 3:**
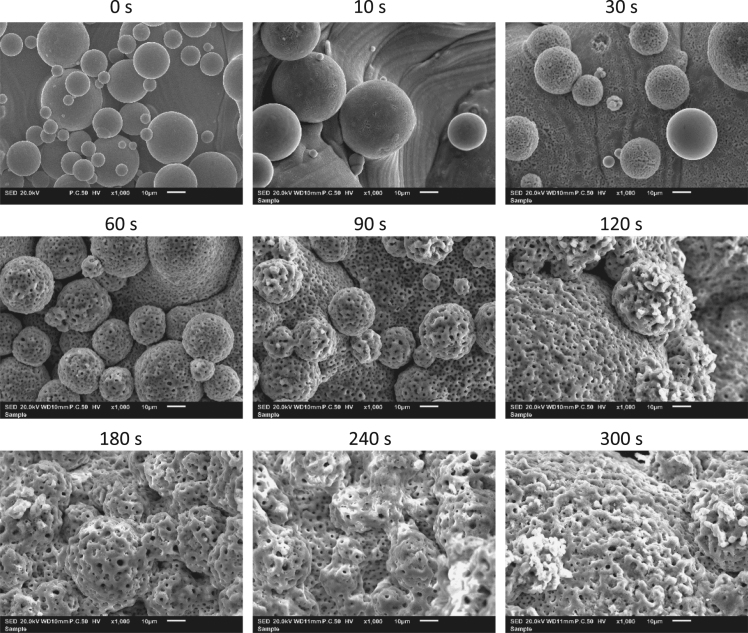
1000-fold magnification scanning electron microscopy images at different time points of Ti-6Al-4V implants treated with plasma electrolytic oxidation.

Ti-6Al-4V implants were synthesized by selective laser melting and subsequently biofunctionalized using the electrochemical plasma electrolytic oxidation process. Scanning electron microscopy was performed at various time points from 0 to 300 s during plasma electrolytic oxidation and is presented in Fig. 1, Fig. 2, Fig. 3. The provided data are part of the experimental results in previous work by the authors [1].

## 2 Experimental design, materials and methods

Ti-6Al-4V implants were manufactured using a selective laser melting printer (SLM-125, Realizer, Borchem, Germany). Specimens were produced from medical grade (grade 23, ELI) Ti-6Al-4V powder (AP&C, Boisbriand, Quebec, Canada) with particle size between 10 and 45 µm and spherical particle morphology. Process parameters were 400 W laser source, layer thickness of 50 µm, laser spot size of 145 µm, layer thickness of 50 µm and exposure time of 300 µs. After selective laser melting, loose powder was removed by vacuum cleaning and samples were thoroughly cleaned by ultrasonication in acetone, 96% ethanol and demineralized water for 5 min subsequently.

Samples were biofunctionalized using plasma electrolytic oxidation on a custom-made laboratory setup. Details regarding the experimental setup can be found in [1]. Plasma electrolytic oxidation is an electrochemical process, which drives uniform growth of the titanium oxide (TiO_2_) layer on the surface of titanium biomaterials. The electrolyte contained 0.02 M calcium glycerophosphate and 0.15 M calcium acetate. Plasma electrolytic oxidation was performed for 0–300 s under galvanostatic conditions.

The surface morphology was analyzed by a scanning electron microscope (JSM-IT100LA, JEOL, Tokyo, Japan). Analysis was performed with beam energies between 5 and 20 kV and a 10 mm working distance. Before analysis, specimens were cleaned for 30 s in acetone and 2-propanol subsequently and coated with a gold layer of 5±2 nm. Imaging was performed at 200, 500 and 1000-fold magnifications (Fig. 1, Fig. 2, Fig. 3).
