# Supplementary material for: Data on the surface morphology of additively manufactured Ti-6Al-4V implants during processing by plasma electrolytic oxidation
Source: Data Brief. 2017 Jun 9;13:385–9. doi: 10.1016/j.dib.2017.06.015 (PMC5480885; doi:10.1016/j.dib.2017.06.015)
Supplement: Supplementary file 1 — Supplementary material [file mmc1.pdf]

Delft University of Technology

Faculty of Mechanical, Maritime & Materials Engineering
Department of Biomechanical Engineering

Address
Mekelweg 2
2628 CD Delft
The Netherlands

To: Prof. Hao-Ran Wang
Editor-in-chief
Data in Brief

Delft, 8 May 2017

Dear Prof Wang,

We are pleased to submit our revised manuscript (DIB_D_17_00196) entitled “Data on the surface morphology of additively manufactured Ti-6Al-4V implants during processing by plasma electrolytic oxidation” by I.A.J. van Hengel, M. Riool, L.E. Fratila-Apachitei, J. Witte-Bouma, E. Farrell, A.A. Zadpoor, S.A.J. Zaat, I. Apachitei together with the responses to the reviewers’ comments.

We would like to thank the reviewers for providing valuable comments that helped us improve the manuscript. We have extensively revised the manuscript along with the reviewers’ comments.

We declare no conflicts of interest with the contents of this manuscript.

Thank you for your cooperation and looking forward to your reply.

Sincerely yours,

Dr. Iulian Apachitei (corresponding author)

Delft University of Technology
Department of Biomechanical Engineering
Mekelweg 2
2628 CD Delft
The Netherlands

Tel +31-15-278 22 76
Fax +31-15-278 47 17
Email i.apachitei@tudelft.nl
